# The Role of Biochemical Cardiac Markers in Atrial Fibrillation

**DOI:** 10.19102/icrm.2023.14101

**Published:** 2023-10-15

**Authors:** Saira Rafaqat, Sana Rafaqat, Hafsa Ijaz

**Affiliations:** 1 Department of Zoology, Lahore College for Women University, Lahore, Pakistan; 2 Department of Biotechnology, Lahore College for Women University, Lahore, Pakistan

**Keywords:** Atrial fibrillation, biochemical cardiac marker, cardiac natriuretic peptide, cardiac troponin, CRP

## Abstract

Atrial fibrillation (AF) is the most common type of cardiac arrhythmia. Proteins are a component of cardiac biomarkers containing cell structures that are released into the circulation when a myocardial injury occurs. They are essential in the diagnosis, risk assessment, and treatment of patients who have chest pain, are thought to have acute coronary syndrome, or are experiencing acute heart failure exacerbations. There are numerous biochemical cardiac markers, but this article summarizes the basic role of major biochemical cardiac markers, including cardiac natriuretic peptides, cardiac troponins, C-reactive protein (CRP), creatine kinase-MB, heart*-*type fatty acid-binding protein, ischemia-modified albumin, lipoprotein (a), osteopontin (OPN), and soluble suppression of tumorigenicity 2 (sST2), in AF. Atrial natriuretic peptide may serve as an indicator of atrial integrity, which may help to select appropriate treatment approaches for AF. Higher levels of N-terminal pro–B-type natriuretic peptide and brain natriuretic peptide are predictive of incidental AF. Increased troponin T release may indicate better clinical results following AF ablation. Similarly, CRP increases the risk of the AF-increasing calcium (Ca) influx in atrial myocytes, but not because of atrial fibrosis. Patients with postoperative AF have lower *FABP3* gene expression in the atrium. Lipoprotein (a) (Lp[a]) may play a causative role in the onset of AF and impact various cardiac tissues. Clinical trials for Lp(a)-lowering drugs should assess their impact on preventing AF. Also, OPN was highly expressed in the circulation of AF patients and further increased with the progression of AF. sST2 was a reliable predictor of new-onset AF and can improve the accuracy of the AF risk model. There is a greater chance that these cardiac biomarkers might be employed to enhance clinical risk stratification in AF.

## Introduction

The cardiac arrhythmia that occurs most frequently is atrial fibrillation (AF). It is a major risk factor for ischemic stroke, which results in severe morbidity and mortality as well as an enormous economic burden. AF affects 37,574 million people globally (0.51% of the population), and its incidence has increased by 33% over the past 20 years. The biggest burden is evident in countries with high socio-demographic indices; however, the most recent increase was reported in countries with moderate socio-demographic indices. By 2050, AF is expected to affect 6–12 million individuals in the United States and 17.9 million people in Europe.^1^ Biomarkers can be molecular, physiologic, biochemical, histologic, or radiographic and may be obtained from serum, body fluid or tissue, whole blood, and plasma.^2–6^ Proteins are a component of cardiac biomarkers containing cell structures that are released into the circulation when a myocardial injury occurs. They are essential in the diagnosis, risk assessment, and treatment of patients who have chest pain, are thought to have acute coronary syndrome (ACS), or are experiencing acute heart failure (HF) exacerbations. In active research, a growing number of unique potential markers have been proposed and incorporated into modern clinical care due to their obvious diagnostic, prognostic, or therapeutic usefulness.^7^ In this context, Pasupathi et al. described numerous biochemical markers in clinical cardiology.^8^ Another study has offered evidence for the presence of traditional cardiac biomarkers.^9^ In their work, Vassiliadis et al. described cardiac markers like creatine kinase-MB (CK-MB), myoglobin, lipoprotein (a) (Lip(a)), brain natriuretic peptide (BNP), troponins I and T (TnI and TnT), osteopontin (OPN), C-reactive protein (CRP), cardiac extracellular matrix, cardiac matrix metalloproteinases, and others,^10^ but this article only emphasizes the role of major biochemical cardiac markers in AF and highlights a few aspects of the pathogenesis of AF, as explained in **Table 1**.

## Methods

Various cardiac biomarkers have been reported in the literature.^8–12^ However, this review article summarizes the basic role of major biochemical cardiac markers—including cardiac natriuretic peptides (NPs), cardiac troponins, CRP, CK-MB, heart-type fatty acid-binding protein (H-FABP), ischemia-modified albumin (IMA), Lip(a), OPN, soluble suppression of tumorigenicity 2 (sST2), lactate dehydrogenase, creatine phosphokinase (CPK), glycogen phosphorylase BB, cardiac myosin light chain kinase, and hydroxybutyrate dehydrogenase—in AF for the first time.

ScienceDirect, PubMed, and Google Scholar were only a few of the databases used to review the literature. January 30, 2023, was the last date of the literature search. Keywords such as “atrial fibrillation,” “biochemical cardiac marker,” “cardiac natriuretic peptides,” “cardiac troponins,” and “CRP” were used. Clinical investigations could only be conducted in English. While we did focus more on current studies, we did not impose any limitations on publication date. It was possible to find related articles by looking through the references of the relevant papers.

### Biochemical cardiac markers in atrial fibrillation

**Figure 1:**
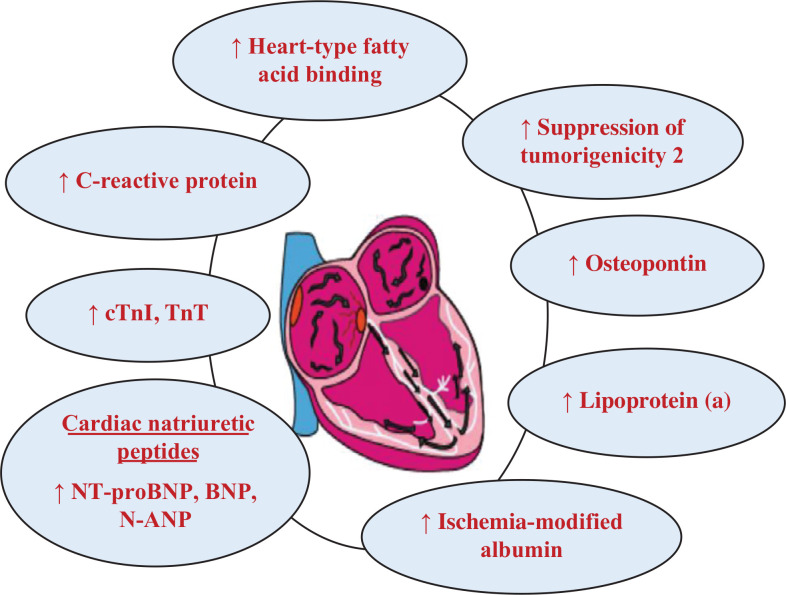
Circulating levels of major cardiac markers in AF subjects. *Abbreviations:* AF, atrial fibrillation; BNP, brain natriuretic peptide; cTnI, cardiac troponin I; N-ANP, N-terminal atrial natriuretic peptide; NT-proBNP, N-terminal pro–B-type natriuretic peptide; TnT, troponin T.

There are numerous cardiac markers, but this article only highlights the major biochemical cardiac markers, including cardiac NPs, cardiac troponins, CRP, CK-MB, H-FABP, IMA, lipoprotein (a), OPN, and sST2, in AF as explained in **Figure 1**.

### Cardiac natriuretic peptides

NPs are a group of structurally related proteins with both natriuretic and diuretic effects. Five distinct peptides make up the NP class, including atrial natriuretic peptide (ANP), BNP, C-type natriuretic peptide, dendroaspis natriuretic peptide, and urodilatin, which is solely generated in the renal district with an autoregulation role.^13^ Heart muscle cells produce ANP, which is a powerful vasodilator and protein (28–amino acid peptide) hormone. ANP is produced in response to atrial distention, stretching of the vessel walls, sympathetic activation of β-adrenoceptors, elevated sodium levels, angiotensin-II, and endothelin.^14^

The atria produce ANPs as a result of atrial stretching. To regulate hemodynamics through natriuresis and vasodilation, acute AF causes atrial stretch, which increases the plasma ANP content. The prolonged AF reduces the atria’s ability to produce ANP by causing structural atrial damage. Thus, it has been determined that the plasma ANP concentration and AF duration have an inverse relationship. A diminished ANP response to exercise has also been demonstrated to be an indicator of unsuccessful cardioversion (CV) of AF to sinus rhythm (SR). Furthermore, a low ANP level was associated with a high atrial collagen concentration, a measure of atrial degeneration. These data suggested that ANP may serve as an indicator of atrial integrity, which might help in the selection of AF patients for treatment approaches.^15^

It may be better to accept accessible biomarkers such as ANP and BNP levels to predict recurrences of AF. The extent of functional atrial myocardium was associated with the production of ANP; both ANP and BNP reflect mechanical stretching as well as atrial pressure, and both are good candidate biomarkers to assess the predisposition to AF recurrence.^16^

Therkelsen et al. described ANP and BNP in AF and discussed alterations to these proteins after CV in persistent AF. Positive correlations between ANP and BNP with left and right atrial volumes were observed. Following CV, changes in left atrial volume were predicted as changes in ANP and BNP. AF might cause continually elevated ANP and BNP, and atrial volume seems to be a significant determinant of ANP and BNP in AF.^17^ Likewise, ANP infusion seems to have an impact on atrial refractoriness, the velocity of conduction, and atrioventricular nodal refractoriness.^18^ Also, Rossi et al. concluded that AF was an independent predictor of elevated N-terminal ANP levels and blurs its link with left ventricular (LV) dysfunction. In contrast, BNP was not independently linked to AF and was strongly determined by LV dysfunction, which is an independent marker.^19^

Higher levels of N-terminal pro–B-type natriuretic peptide (NT-proBNP) and BNP were predictive of incidental AF, while NPs were elevated in AF. The atrial environment may be reflected by NPs, which may help to identify the underlying atrial cardiomyopathy.^20^ Also, Weng et al. concluded that NT-proBNP levels might be helpful as a biomarker to identify a subset of hypertensive individuals who would benefit from intensive blood pressure control or other upstream treatments to target atrial substrate to prevent AF recurrence post-ablation.^21^

NT-proBNP and BNP levels were higher in silent AF/atrial tachycardia patients compared to those with SR. Therefore, NP level–induced Holter monitoring may be a helpful approach in the prevention of stroke or systemic embolism when used to screen for undetected paroxysmal AF.^22^ After CV in AF patients, ANP and BNP dramatically decline, and both can serve as reliable indicators of recurrent AF.^23^ A higher diagnostic threshold should be applied in individuals with AF as the presence of AF was linked to higher circulating BNP levels in patients without HF but not in those with HF.^24^ AF and HF frequently coexist, which is linked to elevated levels of NT-proBNP.

The diagnostic accuracy of NT-proBNP for HF can be impaired by AF. In stable outpatients with cardiovascular risk factors, NT-proBNP was a more accurate biomarker for incident and prevalent AF than for HF. It may not be useful to diagnose chronic HF, especially HF with preserved ejection fraction, in individuals with AF. If NT-proBNP levels were higher without evidence of HF, screening for AF should be taken into account.^25^

Moreover, it has been suggested that secretory granules in the atria release higher levels of BNP during AF, and BNP elevation of unknown cause may be associated with the presence of asymptomatic AF. Cardiac function assessment using BNP during AF requires special consideration, unlike during SR, even in patients with paroxysmal AF or chronic AF, because BNP level during AF is the sum of BNP values from the ventricle (reflecting LV function) and the atrium (due to AF).^26^

Shin et al. concluded that elevated plasma levels of NT-proBNP were related to AF in individuals with a normal left ventricular ejection fraction (LVEF), and these levels drop when electrical CV may restore a sustained SR. However, when AF recurs, NT-proBNP appears to rise (albeit not significantly). Finally, NT-proBNP was not a reliable indicator of the long-term effectiveness of SR restoration by electrical CV.^27^ In contrast, Brady et al. explained that AF patients had elevated NT-proBNP concentrations, which predicted future HF events irrespective of the occurrence of HF, thus encouraging regular measurement of NT-proBNP in the assessment of patients with AF.^28^ Also, measuring NT-proBNP is a promising technique that can help clinicians in choosing a rhythm-control or rate-control approach for managing AF.^29^

### Cardiac troponins

Troponin is a complex made up of a trio of single-chain polypeptides: troponin-1 (Tn-1), which stops muscle contraction in the absence of calcium (Ca); TnT, which binds the troponin complex to tropomyosin; and troponin C, which binds Ca. Troponin controls muscle contractions together with tropomyosin, which is under the control of Ca. Within 3–6 h following a myocardial infarction (MI), the cardiac muscle–specific isoform C Tn-1 levels increase. Within 14–20 h, the levels reach their peak, then return to normal after 5–7 days.^11^

The recommended biomarker for detecting myocardial injury is troponin. Elevated troponin levels are very specific for cardiac damage, and there are several critical difficulties relating to the fundamental science of this protein and its measurement. Hence, understanding how sensitive the specific test is for diagnosing cardiac injury is necessary.^8^

Piechota et al. investigated the factors that lead to the elevation of cardiac TnI (cTnI) and explored the possibility that an external CV of AF may induce this elevation. A high-sensitivity experiment therefore demonstrated that CV can cause a small but significant rise in cTnI. The impact of CV on cTnI elevation appears to be more apparent in individuals with LV end-diastolic dimension that was rather severe.^30^ On the contrary, Knayzer et al. detailed that, after cardiac surgery, a strong relationship was found between elevation of the cTnI plasma level and clinical inflammation–associated parameters. These variables were not correlated with postoperative AF (POAF), nor did postoperative plasma cTnI levels correlate with the occurrence of AF. Although more research is needed, preoperative statin therapy might help to reduce the postoperative inflammatory response.^31^ Also, the TnI level was not linked to the development of AF, according to the study by Zhang et al., but another biomarker of myocardial necrosis, the CK-MB level, was found to be predictive of AF development. Longer alterations in the myocardial structure may cause more marked heart damage and an increased risk of AF.^32^

As shown by high-sensitivity TnT measurement, external direct-current CV did not cause myocardial damage within the first 6 h. Patients who have had CV and whose cardiac troponin levels increase significantly thereafter should be examined for other potential causes of myocardial injury rather than being automatically concluded to have suffered myocardial injury as a result of the CV.^33^ Similar to this, Yoshida et al. found that increased TnT release can predict better clinical results following AF ablation. The authors hypothesized that a greater increase in TnT following ablation signifies the existence of a healthier left atrium, thereby correlating TnT release with better clinical outcomes.^34^

Among AF patients, high-sensitivity troponin levels were greater in men than in women. In anticoagulated individuals with AF, however, high-sensitivity troponin concentrations remain equally related to poor clinical outcomes regardless of sex.^35^ In many stable individuals with persistent AF who did not have ischemic heart disease, elevated TnT was found. A substantial decrease in heart rate due to the study drugs was connected to a large drop in TnT level.^36^

Troponin elevation was frequent in individuals who report to hospitals with acute symptomatic AF, but it was not a good predictor of underlying significant coronary artery disease in this patient population. The presence of severe coronary artery disease, however, was predicted by the cTnI peak.^37^ Patients with high heart rates, angina pectoris, and/or AF frequently have inaccurately inflated cTnI values. Clinicians examining individuals with acute AF and symptoms of myocardial ischemia should consider these results. It is necessary to make appropriate clinical recommendations that take into account AF-related elevations in cTnI.^38^

In a sizable “real-world” cohort of anticoagulated AF patients, both high-sensitivity TnT and high-sensitivity interleukin (IL)-6 levels provided prognostic information, enhancing clinical risk scores for the prediction of long-term cardiovascular events and mortality. According to the authors’ findings, there was a greater chance that these biomarkers might be employed to enhance clinical risk stratification in AF.^39^ Elevated TnI and NT-proBNP levels were frequently present in AF patients, with each being individually associated with an increased risk of stroke and death. Beyond frequently employed clinical indicators, cardiac biomarkers are effective for enhancing risk prediction in AF.^40^

A high-sensitivity cTnI level was not able to enhance risk stratification but was an independent prognostic factor for the occurrence of AF hospitalization in a community-based population.^41^ AF or atrial flutter electrical CV results in an increase in total CK but no change in cardiac TnT, suggesting the absence of myocardial damage and that CK was derived from skeletal muscle. Following CV for atrial arrhythmias, if cardiac TnT increases, it is important to investigate other causes of myocardial injury.^42^

### C-reactive protein

CRP is a typical biomarker of systemic inflammation linked to several cardiovascular risks and involved in the immunological process that results in cardiac and vascular remodeling. Although a Mendelian randomization analysis did not support the causative effects of CRP on AF, evidence from observational studies revealed that increased blood CRP levels were positively linked to the incidence of AF.^43^ Similarly, Chang et al. explained that CRP greatly increased the inward L-type Ca current in atrial myocytes while not affecting the production of pro-collagen genes in atrial fibroblasts or other ionic currents. This suggests that CRP increases the risk of the AF-increasing Ca influx in atrial myocytes, but not because of atrial fibrosis.^44^ Meyre et al. indicated that inflammation is crucial to the development and progression of AF. However, little is known about the connection between subclinical inflammation and the recurrence of AF following catheter ablation. As a result, in patients undergoing catheter ablation for AF, greater pre-interventional CRP levels were linked to a higher risk of AF recurrence.^45^

Plasma high-sensitivity CRP (hs-CRP) concentration before circumferential pulmonary vein isolation (CPVI) was linked to AF recurrence after a primary CPVI procedure in both paroxysmal and persistent AF patients. The plasma hs-CRP concentration might play a role in the prediction of AF recurrence after primary CPVI.^46^ In the same context, Sotomi et al. showed that abnormally high preoperative CRP levels were a reliable indicator of very late recurrence (VLR) after AF ablation. In clinical practice, ongoing monitoring for VLR following pulmonary vein isolation was preferable, especially in the cohort of patients who were at high risk for VLR.^47^

In AF patients, CRP may not only be a measure of inflammation but also actively participate in pathophysiology. In contrast, CRP might be more of a result than a cause of the pathophysiology of AF. Epidemiologic studies can find relationships but not causality, which needs to be established by other clinical research. The use of a straightforward marker for the assessment of inflammatory activity in AF takes up new possibilities for the development of medications that will influence inflammation (aspirin, statins, fibrates, angiotensin inhibitors, etc.).^48^

Ock et al. concluded that increased plasma CRP concentrations may be an accurate surrogate measure for assessing the degree of carotid atherosclerosis in patients with AF, and CRP concentration may also be associated with an increased risk of ischemic stroke. AF patients with elevated CRP levels were more likely to experience advanced atherosclerosis and cerebrovascular accidents. These patients might require anti-inflammatory treatments, such as statins and anticoagulants. The findings should be investigated in more detail in laboratory, observational, and interventional studies.^49^

A dose-dependent link was found between postoperative serum CRP level and the emergence of POAF in people who have had undergone coronary artery bypass graft surgery. The percentage of patients with POAF in the first serum CRP quartile (24.5% in CRP ≤ 90 mg/L quartile) and the fourth quartile (35.1% in CRP > 175 mg/L quartile) differed in statistically significant fashion.^50^

According to another study, monitoring serum CRP both pre- and postoperatively may help predict the development of POAF.^51^ More research is required to determine the value of CRP and other inflammatory markers in the prediction of POAF, rather than simply examining the relationship between CRP and POAF/AF. Future research must consider the many variables that could affect CRP, including comorbidities, age, and infections.^52^

In long-standing persistent/permanent AF patients, inflammatory infiltrates, blood levels of CK-MB and CRP, and their ages were all significantly increased. In addition, in both AF subtypes, CRP blood levels and the degree of atrial inflammation tended to be moderately positively correlated.^53^ It has been demonstrated that CRP blood levels were elevated in individuals with AF and that they positively correlated with the diameter and duration of AF in paroxysmal^54^ as well as persistent and chronic AF.^55^

The study by Narducci et al. showed the viability and safety of a novel method for collecting atrial samples during a typical transseptal puncture. Paroxysmal AF was more likely than persistent AF to be caused by local inflammation, as determined by CRP localization in atrial tissue.^56^ After successful CV, hs-CRP assays were moderately accurate in predicting the recurrence of AF.^57^ Increased CRP levels could be a sign of an inflammatory condition that promotes AF development.^58^

A two-fold elevation in CRP was linked to AF. The largest CRP elevation was found in individuals who had more persistent AF, which may indicate that CRP is a marker of inflammatory conditions that may encourage the persistence of AF, possibly by causing structural and/or electrical remodeling of the atria. These pathways may signify a novel mechanism by which structural changes resulting from inflammation perpetuate AF. These findings need to be tested and verified in a larger trial. However, these pathways could offer a potential target for pharmacological disruption or reversal of atrial structural remodeling. The pharmaceutical therapies for AF that are now available have a poor success rate and possibly harmful side effects. For innovative, more tolerated pharmacological AF treatments, inflammatory pathways may serve as a basis. It may be necessary to conduct randomized studies on agents such as anti-inflammatory medications and/or other CRP-lowering drugs.^59^

### Creatine kinase–MB

An enzyme called creatine kinase is made up of a pair of subunits, M and/or B. Three separate isoenzymes, CK-BB, CK-MB, and CK-MM, are produced when a trio of different pairings of these units join. The heart-specific isoenzyme CK-MB has long served as the gold standard for diagnosing acute MI in laboratories. Although it is mostly cardiac-specific and may also be found in skeletal muscles and other tissues, it is present in larger amounts in the heart muscle.^60^

NT-proBNP, CK-MB, and LVEF are the three main prognostic factors utilized to predict new-onset AF in ACS patients. The value suitable for AF screening can be quantitatively determined using NT-proBNP and CK-MB.^32^ In contrast, CPK-MB and AF were shown to not be correlated in the investigation by Jeppesen et al. Patients with POAF had a statistically significant 2.4-fold higher incidence of paroxysmal or permanent AF than those with postoperative SR. Postoperative CPK-MB was not shown to be a valid indicator of late cardiac mortality or the identification of paroxysmal or persistent AF within 10 years of coronary artery bypass graft surgery. Patients who experienced POAF had a greater risk of acquiring paroxysmal or chronic AF later than those who experienced postoperative SR.^61^

### Heart-type fatty acid–binding protein

The heart-type fatty acid–binding protein family includes H-FABP, which is also referred to as a mammary-derived growth inhibitor.^62^ The myocardial fatty acid transporter known as H-FABP is released into the bloodstream following myocardial damage. Following elective CV for AF, H-FABP was not significantly altered, leading to the hypothesis that myocardial necrosis had not taken place during CV.^63^

After cardiac surgery, there was a greater increase in H-FABP, which suggests that ischemia myocardial injury was one of the underlying mechanisms that contribute to AF. POAF may be less frequent as a result of measures taken to reduce perioperative ischemia damage.^64^ Patients with POAF have lower *FABP3* gene expression in the atrium. The authors’ findings suggested a potential connection between altered fatty acid transport in the atrium and elevated AF onset after cardiac surgery.^65^

### Ischemia-modified albumin

IMA is a marker of myocardial ischemia measured with the albumin cobalt binding test. In the same context, to ascertain if temporary myocardial ischemia happens following elective direct current CV for AF, Roy et al. assessed IMA concentrations. In comparison to those without these modifications, patients with electrocardiographic abnormalities (ST depression and/or T-wave inversion) following CV had substantially greater levels of IMA. As a result, myocardial ischemia that is temporary may be reflected by higher levels of IMA following CV.^66^

### Lipoprotein (a)

With an additional apolipoprotein (a) (apo[a]) joined to the apolipoprotein (b) component of the low-density lipoprotein (LDL) particle by a disulfide bridge, Lp(a) is an LDL particle. Due to the wide variety of apo(a) isoforms present in the population, the structure of Lp(a) is quite diverse. Etiologically, promoting wound healing and minimizing bleeding, particularly during childbirth, may have given a survival advantage.^67^ High levels of Lp(a) are a direct cause and an independent risk factor for cardiovascular diseases (CVDs) in epidemiological and genetic investigations. Both pro-atherogenic and prothrombotic pathways may play a role in the elevated risk of CVD caused by Lp(a).^68^

The lower incidence rates of AF are linked to greater levels of Lp(a). It might be useful to perform a genomic analysis to ascertain whether gene scores for Lp(a) levels are related to the risk of AF.^69^ In contrast, high Lp(a) levels are not linked to the occurrence of AF. Lp(a) levels are primarily related to a higher risk of ischemic stroke in those without AF but not in those with AF.^70^

Another study reported that Lp(a) may play a causative role in the onset of AF and impact various cardiac tissues. Clinical trials for Lp(a)-lowering drugs should assess their impact on preventing AF.^71^ Although several studies have attempted to demonstrate a potential association between AF and high Lp(a) levels, the authors were unable to find such a relationship. However, when compared to other ethnic populations, ethnic variations might partially account for these findings in patients.^72^

Thromboembolism is more likely to occur in those with persistent AF. Because apo(a) and plasminogen have a structural similarity, Lp(a) might induce thrombosis by modifying the fibrinolytic system. Strong associations exist between left atrial thrombus and elevated blood levels of Lp(a). These results imply that Lp(a) level may represent a potential risk factor for left atrial thrombus in individuals with persistent AF.^73^

### Osteopontin

The human OPN protein has 314 amino acid residues, and its predicted molecular weight is 32 kDa. A few of the cell types in the heart that produce OPN in response to various stimuli, such as hypoxia, inflammation, toxin exposure, and mechanical stretching, include cardiomyocytes, cardiac fibroblasts, resident macrophages, and coronary artery endothelial cells.^74^ OPN is an extracellular matrix protein that plays an integral role in myocardial remodeling and has previously been shown to be a valuable biomarker in CVD.^75^ OPN, a plasma-detectable glycoprotein found to be elevated in various animal models of cardiac failure, may therefore constitute a new biomarker that makes it simpler for patients with HF to categorize their risks.^76^

A common feature of AF is atrial fibrosis. According to recent reports, OPN can induce fibrosis in the kidneys, liver, and lungs. Lin et al. reported that OPN is highly expressed in the circulation of AF patients and further increased with the progression of AF. Additionally, correlation analysis revealed a significant relationship between circulating OPN and low-voltage areas, which is a marker of atrial fibrosis in AF patients. Immunohistological staining and immunoblotting showed that AF patients with more severe atrial fibrosis have greater levels of OPN expression. OPN increased fibroblast proliferation and elevated the production of both collagen I and fibronectin according to in vitro investigations in cultured human atrial fibroblasts. The profibrotic effects of OPN on atrial fibroblasts were produced via stimulating the Akt/glycogen synthase kinase-3β (GSK-3β)/β-catenin pathway and inhibiting autophagy, respectively.^77^

OPN is also known as secreted phosphoprotein 1 (SPP1), which has been implicated in the fibrosis process, including in atrial fibrosis. Du et al. provided the suggestion that SPP1 contributes to mitochondrial DNA damage and the resultant atrial fibrosis. The underlying regulatory mechanism here is that SPP1 exerts its function by acting as a promoting factor for transforming growth factor-β (TGF-β) and increasing the activity of sterol-regulatory element binding protein-2 (SREBP2)/proprotein convertase subtilisin/kexin type 9 (PCSK9), thus forming an SPP1/TGF-β/SREBP2/PCSK9 axis to participate in atrial fibrosis. These findings provide a new molecular theoretical basis for the understanding of the pathogenesis of atrial fibrosis.^78^

Moreover, it has been recognized that polo-like kinase 2 (PLK_2_) is an epigenetically regulated kinase involved in the pathogenesis of fibrosis in AF. PLK_2_ knockout mice can serve as a model of diastolic HF wherein OPN is a promising therapeutic target. The authors’ findings support the idea that AF is a complex systemic condition as well as an ion channel disease. To prevent and treat fibrosis and diastolic HF in AF, it may be beneficial to use 5-aminosalicylic acid to limit OPN release and restore physiological PLK_2_ expression.^79^ Similarly, Tipteva et al. reported that serum OPN levels were independently linked to AF in aortic valve stenosis patients, leading to speculation about its predominant profibrotic function in the left atrium.^80^

OPN has been described as a novel, independent risk factor for AF.^81^ The rate of recurrence after AF catheter ablation is exacerbated by atrial fibrosis. OPN is a multifunctional molecule that plays a role in fibrosis and other pathological pathways. Also, Güneş et al. showed that, in patients undergoing cryoballoon AF ablation, both the persistence of AF and high pre-procedural OPN levels independently predict AF recurrence.^82^

### Soluble suppression of tumorigenicity 2

ST2 is an IL-1 receptor family member and has been identified as a novel biomarker for cardiac strain. In patients with chronic HF, elevated sST2 concentrations predict sudden cardiac death and offer additional data on NT-proBNP levels. The clinical decision-making process may be impacted by a combined biomarker strategy.^83^ Recently, ST2 has gained interest as a possible biomarker in various fields. It contributes to cardiovascular pathophysiology and is implicated in a variety of inflammatory disorders and allergies. ST2 is now being studied as a potential biomarker for cardiac disorders. A cardioprotective pathway that reduces inflammatory response, hypertrophy, and death of cardiomyocytes includes the interaction of IL-33 and ST2L. Clinical research suggests that sST2 may be effective in treating several diseases, including arrhythmia, hypertension, myocarditis, acute aortic syndrome, and coronary artery disease. The perioperative period and heart transplantation may both benefit from the use of this novel biomarker.^84^ Also, sST2 is an independent predictor of death or HF in patients with AF irrespective of a history of HF or the NT-proBNP level.^85^

In another study, Tan et al. concluded that circulating sST2 ≥ 39.25 ng/mL in patients with persistent AF predicts recurrence after initial ablation. Additionally, atrial myofibroblasts are probably a biological source of circulating sST2, which might be a significant biomarker for the severity of atrial fibrosis. To enhance the results of catheter ablation for persistent AF, inhibiting circulating sST2 may be helpful as adjuvant therapy.^86^ Even after accounting for additional clinical variables, ST2 and BNP continue to function as independent predictors of AF. Elevated BNP seems to be a better predictor of AF than ST2 when ST2 is combined with it.^87^

The study conducted by Okar et al. is the first research study to link sST2 to the recurrence of AF. For predicting AF recurrence, sST2 was found to be the sole independent parameter.^88^ A promising diagnostic for identifying individuals with high-grade fibrosis who may benefit less from cryoablation is likely to be the atrial fibrosis–related protein sST2. Many ablation operations or different types of ablation techniques could be used for such patients.^88^

Wang et al. showed that individuals with paroxysmal AF and low-voltage zones of >20% had higher sST2 levels than patients with smaller low-voltage zones. The authors also revealed that elevated sST2 levels might act as a unique predictor of AF recurrence rate in individuals who have undergone radiofrequency catheter ablation.^89^ Likewise, in individuals with normal LV systolic function, sST2 but not galectin-3 can be used to predict SR maintenance following CV of AF.^90^

sST2 and tissue inhibitor of metalloproteinase-1 (TIMP-1) were linked to the development of AF in a cohort analysis, regardless of clinical traits or biomarkers. Age, increased NT-proBNP, an enlarged left atrium, and sST2 and TIMP-1 all worked together to provide a reliable indicator of AF development.^91^ Also, in individuals with acute MI, sST2 was a reliable predictor of new-onset AF and can improve the accuracy of the AF risk model.^92^

According to Kong et al., an elevation in serum ST2 concentration may contribute to the pathophysiological mechanisms underlying the onset of AF; specifically, this increase is associated with atrial remodeling, which suggests that increased serum ST2 levels could serve as a biomarker for assessing the severity of AF and predicting the likelihood of recurrent episodes, particularly in patients with persistent AF following treatment.^93^

Independent of left atrial size, sST2 is a marker of advanced functional remodeling in AF patients that has been linked to much longer ablation treatments. Future research is necessary to determine whether elevated sST2 levels indicate an increased risk of atrial arrhythmia recurrence after ablation.^94,95^

The objective biomarker sST2 is probably capable of predicting the probability of emergency hospitalization or HF in AF patients. Increased sST2 levels may contribute to the development of AF.^96^ Both sST2 and left atrial diameter have been identified as independent predictors of HF by Cox proportional hazard analysis. In comparison to SR, AF correlates with greater sST2 concentrations. In individuals with AF, plasma sST2 may be a valuable biomarker for anticipating HF.^97^

Atrial stretch is a well-known factor in the development of AF, which is caused by pathophysiologically elevated hemodynamic stress; it may also promote the release of sST2 and BNP. Because AF patients have a faster heart rate and atrial pressure than SR patients, this may be the reason why sST2 levels increased during AF. Stretched cardiac fibroblasts and cardiomyocytes were subject to biomechanical overload, and ST2/IL-33 signaling was assumed to be crucial in controlling the myocardial response. Loss of IL-33/ST2L signaling causes excessive myocyte hypertrophy, fibrosis, impairment of LV function, and an increased risk of mortality from ventricular failure, which all contribute to remodeling of the ventricular myocardium.^98^

## Conclusion

This review article concludes that biochemical cardiac markers, including cardiac NPs, cardiac troponins, CRP, CK-MB, H-FABP, IMA, Lp(a), OPN, and sST2, play a significant role in the pathogenesis of AF, as explained in **Table 1**. Additional studies are required to find the link between other cardiac markers and AF. Furthermore, strategies for treatment are necessary to establish the early management of these cardiac markers, which will ultimately decrease the prevalence of cardiac arrhythmia disorders.

## Figures and Tables

**Table 1:** Role of Major Biochemical Cardiac Markers in the Pathogenesis of AF

Biochemical Cardiac Markers	Pathogenesis of AF
NPs	Both ANP and BNP reflect mechanical stretching as well as atrial pressure, and both are good candidate biomarkers to assess the predisposition to AF recurrences. ANP infusion seems to have an impact on atrial refractoriness, the velocity of conduction, and atrioventricular nodal refractoriness. During AF, atrium enlargement and atrial pressure increases are linked to raised plasma concentration of ANP. Increased ANP levels during an AF episode are probably an acute physiological response to increased atrial pressure.
CRP	CRP greatly increase the inward L-type Ca current in atrial myocytes while not affecting the production of pro-collagen genes in atrial fibroblasts or other ionic currents. This suggests that CRP increases the risk of the AF-increasing Ca influx in atrial myocytes, but not because of atrial fibrosis. CRP is a marker of inflammatory conditions that may encourage the persistence of AF, possibly by causing structural and/or electrical remodeling of the atria. Increased CRP levels could be a sign of an inflammatory condition that promotes AF development.
H-FABP	After cardiac surgery, there is a greater increase in H-FABP, which suggests that ischemia myocardial injury is one of the underlying mechanisms that contribute to AF. Patients with POAF have lower *FABP3* gene expression in the atrium. These results raise the possibility that increased AF onset following cardiac surgery may be related to altered fatty acid transport in the atria.
IMA	An increase in IMA was greater in individuals with ST-T electrocardiographic alterations than in those without such changes, indicating that myocardial ischemia may be a potential cause for the development of these changes.
Lp(a)	Lp(a) is a potential causal mediator in the development of AF, which shows that the effects of Lp(a) extend across myocardial tissues. Lp(a) level may represent a potential risk factor for left atrial thrombus in individuals with persistent AF.
OPN	OPN was highly expressed in the circulation of AF patients. Circulating OPN is positively related to LVAs (a marker of atrial fibrosis) in AF patients. OPN promotes the proliferation of fibroblasts and increases the production of collagen I and fibronectin. The profibrotic effects of OPN on atrial fibroblasts are determined via activating Akt/GSK-3β/β-catenin signaling and suppressing autophagy. Serum OPN levels have a predominant profibrotic role in the left atrium. OPN contributes to mtDNA damage and the resultant atrial fibrosis. To prevent and treat fibrosis and diastolic HF in AF, it may be beneficial to use 5-ASA to limit osteopontin release and restore physiological PLK_2_ expression.
sST2	sST2, which is a circulating biomarker for the degree of atrial fibrosis, is most likely produced by atrial myofibroblasts. Baseline sST2 is able to predict recurrence following ablation in individuals with persistent AF and has a favorable correlation with LAVI. sST2, which is related to atrial fibrosis, might be a useful marker for the detection of patients with high-grade fibrosis who will get less benefit from cryoablation. An increase in serum ST2 concentration may play a significant pathophysiological role in the onset of AF and is related to atrial remodeling. sST2 is a reliable predictor of new-onset AF and can improve the accuracy of the AF risk model.

*Abbreviations:* 5-ASA, 5-aminosalicylic acid; AF, atrial fibrillation; ANP, atrial natriuretic peptide; BNP, brain natriuretic peptide; Ca, calcium; CRP, C-reactive protein; GSK-3β, glycogen synthase kinase-3β; HF, heart failure; H-FABP, heart-type fatty acid-binding protein; IMA, ischemia-modified albumin; LAVI, left atrial volume index; Lp(a), lipoprotein (a); mtDNA, mitochondrial DNA; NP, natriuretic peptide; OPN, osteopontin; PLK_2_, polo-like kinase 2; POAF, postoperative atrial fibrillation; sST2, soluble suppression of tumorigenicity 2.

## References

[1] Lippi G, Sanchis-Gomar F, Cervellin G (2021). Global epidemiology of atrial fibrillation: an increasing epidemic and public health challenge. Int J Stroke.

[2] Stern AD, Alexander BM, Chandra A (2018). Innovation incentives and biomarkers. Clin Pharmacol Ther.

[3] Aydin S, Aydin S, Patel VB, Preedy VR (2016). Irisin concentrations as a myocardial biomarker. Biomarkers in Cardiovascular Disease.

[4] Khan IA, Wattanasuwan N (2005). Role of biochemical markers in diagnosis of myocardial infarction. Int J Cardiol.

[5] Wu AH, Apple FS, Gibler WB, Jesse RL, Warshaw MM, Valdes R (1999). National academy of clinical biochemistry standards of laboratory practice: recommendations for the use of cardiac markers in coronary artery diseases. Clin Chem.

[6] Aydin S, Ugur K, Aydin S, Sahin İ, Yardim M (2019). Biomarkers in acute myocardial infarction: current perspectives. Vasc Health Risk Manag.

[7] Singh V, Martinezclark P, Pascual M, Shaw ES, O’Neill WW (2010). Cardiac biomarkers – the old and the new: a review. Coron Artery Dis.

[8] Pasupathi P, Rao YY, Farook J, Bakthavathsalam G (2009). Biochemical cardiac markers in clinical cardiology. J Med.

[9] Parsanathan R, Jain SK (2020). Novel invasive and noninvasive cardiac-specific biomarkers in obesity and cardiovascular diseases. Metab Syndr Relat Disord.

[10] Vassiliadis E, Barascuk N, Didangelos A, Karsdal MA (2012). Novel cardiac-specific biomarkers and the cardiovascular continuum. Biomark Insights.

[11] Padmaja V, Deepu P (2009). Cardiac biomarkers. Hygeia J Drugs Med.

[12] Rafaqat S, Afzal S, Rafaqat S, Khurshid H, Rafaqat S (2022). Cardiac markers: role in the pathogenesis of arterial hypertension. World J Hypertens.

[13] Vesely DL, Douglass MA, Dietz JR (1994). Three peptides from the atrial natriuretic factor prohormone amino terminus lower blood pressure and produce diuresis, natriuresis, and/or kaliuresis in humans. Circulation.

[14] Ahmed F, Tabassum N, Rasool S (2012). Regulation of atrial natriuretic peptide (ANP) and its role in blood pressure. Int Curr Pharm J.

[15] Van Den Berg MP, van Gelder IC, van Veldhuisen DJ (2004). Depletion of atrial natriuretic peptide during longstanding atrial fibrillation. Europace.

[16] Zografos TA, Katritsis DG (2013). Natriuretic peptides as predictors of atrial fibrillation recurrences following electrical cardioversion. Arrhythm Electrophysiol Rev.

[17] Therkelsen SK, Groenning BA, Kjaer A, Svendsen JH, Jensen GB (2008). ANP and BNP in atrial fibrillation before and after cardioversion – and their relationship to cardiac volume and function. Int J Cardiol.

[18] Crozier I, Richards AM, Foy SG, Ikram H (1993). Electrophysiological effects of atrial natriuretic peptide on the cardiac conduction system in man. Pacing Clin Electrophysiol.

[19] Rossi A, Enriquez-Sarano M, Burnett JC, Lerman A, Abel MD, Seward JB (2000). Natriuretic peptide levels in atrial fibrillation: a prospective hormonal and Doppler-echocardiographic study. J Am Coll Cardiol.

[20] Kerr B, Brandon L (2022). Atrial fibrillation, thromboembolic risk, and the potential role of the natriuretic peptides, a focus on BNP and NT-proBNP – a narrative review. Int J Cardiol Heart Vasc.

[21] Weng W, Choudhury R, Sapp J (2021). The role of brain natriuretic peptide in atrial fibrillation: a substudy of the substrate modification with aggressive blood pressure control for atrial fibrillation (SMAC-AF) trial. BMC Cardiovasc Disord.

[22] Seegers J, Zabel M, Grüter T (2015). Natriuretic peptides for the detection of paroxysmal atrial fibrillation. Open Heart.

[23] Zeng QX, Wei MF, Zhang W, Zhang Y, Zhong JQ (2010). Level of natriuretic peptide determines outcome in atrial fibrillation. J Atr Fibrillation.

[24] Knudsen CW, Omland T, Clopton P (2005). Impact of atrial fibrillation on the diagnostic performance of B-type natriuretic peptide concentration in dyspneic patients: an analysis from the breathing not properly multinational study. J Am Coll Cardiol.

[25] Werhahn SM, Becker C, Mende M (2022). NT-proBNP as a marker for atrial fibrillation and heart failure in four observational outpatient trials. ESC Heart Fail.

[26] Tsuchida K, Tanabe K (2004). Influence of paroxysmal atrial fibrillation attack on brain natriuretic peptide secretion. J Cardiol.

[27] Shin DI, Jaekel K, Schley P (2005). Plasma levels of NT-pro-BNP in patients with atrial fibrillation before and after electrical cardioversion. Z Kardiol.

[28] Brady PF, Chua W, Nehaj F (2022). Interactions between atrial fibrillation and natriuretic peptide in predicting heart failure hospitalization or cardiovascular death. J Am Heart Assoc.

[29] Marsiliani D, Buccelletti F, Carroccia A, Gilardi E, Silveri NG, Franceschi F (2010). Natriuretic peptides and atrial fibrillation. Eur Rev Med Pharmacol Sci.

[30] Piechota W, Gielerak G, Ryczek R, Kaźmierczak A, Bejm J, Piechota W (2007). Cardiac troponin I after external electrical cardioversion for atrial fibrillation as a marker of myocardial injury – a preliminary report. Kardiol Pol.

[31] Knayzer B, Abramov D, Natalia B, Tovbin D, Ganiel A, Katz A (2007). Atrial fibrillation and plasma troponin I elevation after cardiac surgery: relation to inflammation-associated parameters. J Card Surg.

[32] Zhang H, Dong P, Yang X (2020). Prognostic indicators of new onset atrial fibrillation in patients with acute coronary syndrome. Clin Cardiol.

[33] Lobo R, Jaffe AS, Cahill C (2018). Significance of high-sensitivity troponin T after elective external direct current cardioversion for atrial fibrillation or atrial flutter. Am J Cardiol.

[34] Yoshida K, Yui Y, Kimata A (2014). Troponin elevation after radiofrequency catheter ablation of atrial fibrillation: relevance to AF substrate, procedural outcomes, and reverse structural remodeling. Heart Rhythm.

[35] Røsjø H, Hijazi Z, Omland T (2020). Cardiac troponin is associated with cardiac outcomes in men and women with atrial fibrillation, insights from the ARISTOTLE trial. J Intern Med.

[36] Ulimoen SR, Enger S, Norseth J (2014). Improved rate control reduces cardiac troponin T levels in permanent atrial fibrillation. Clin Cardiol.

[37] Alghamry A, Hanna J, Pelecanos A (2016). Predictors of significant coronary artery disease in atrial fibrillation: are cardiac troponins a useful measure. Int J Cardiol.

[38] Parwani AS, Boldt LH, Huemer M (2013). Atrial fibrillation-induced cardiac troponin I release. Int J Cardiol.

[39] Roldan V, Marin F, Diaz J (2012). High sensitivity cardiac troponin T and interleukin-6 predict adverse cardiovascular events and mortality in anticoagulated patients with atrial fibrillation. J Thromb Haemost.

[40] Hijazi Z, Oldgren J, Andersson U (2012). Cardiac biomarkers are associated with an increased risk of stroke and death in patients with atrial fibrillation: a randomized evaluation of long-term anticoagulation therapy (RE-LY) substudy. Circulation.

[41] Zhu K, Hung J, Divitini M (2018). High-sensitivity cardiac troponin I and risk of incident atrial fibrillation hospitalisation in an Australian community-based cohort: the Busselton health study. Clin Biochem.

[42] Greaves K, Crake T (1998). Cardiac troponin T does not increase after electrical cardioversion for atrial fibrillation or atrial flutter. Heart.

[43] Li X, Peng S, Wu X (2022). C-reactive protein and atrial fibrillation: Insights from epidemiological and Mendelian randomization studies. Nutr Metab Cardiovasc Dis.

[44] Chang SN, Tsai CT, Wu CK (2012). A functional variant in the promoter region regulates the C-reactive protein gene and is a potential candidate for increased risk of atrial fibrillation. J Intern Med.

[45] Meyre PB, Sticherling C, Spies F (2020). C-reactive protein for prediction of atrial fibrillation recurrence after catheter ablation. BMC Cardiovasc Disord.

[46] Liu J, Fang PH, Dibs S, Hou Y, Li XF, Zhang S (2011). High-sensitivity C-reactive protein as a predictor of atrial fibrillation recurrence after primary circumferential pulmonary vein isolation. Pacing Clin Electrophysiol.

[47] Sotomi Y, Inoue K, Ito N (2013). Incidence and risk factors for very late recurrence of atrial fibrillation after radiofrequency catheter ablation. Europace.

[48] Madrid AH (2006). C-reactive protein and atrial fibrillation. An old marker looking for a new target. Rev Esp Cardiol.

[49] Ock SY, Cho KI, Kim HJ (2013). The impacts of C-reactive protein and atrial fibrillation on carotid atherosclerosis and ischemic stroke in patients with suspected ischemic cerebrovascular disease: a single-center retrospective observational cohort study. Korean Circ J.

[50] Olesen OJ, Vinding NE, Østergaard L (2020). C-reactive protein after coronary artery bypass graft surgery and its relationship with postoperative atrial fibrillation. Europace.

[51] Tanaka M, Imano H, Kubota Y (2021). Serum high-sensitivity C-reactive protein levels and the risk of atrial fibrillation in Japanese population: the circulatory risk in communities study. J Atheroscler Thromb.

[52] Xie JY, Noeman M, Pimenta D, Little C (2021). C-reactive protein as a predictor for developing post-operative atrial fibrillation. Europace.

[53] Wu L, Emmens RW, van Wezenbeek J (2020). Atrial inflammation in different atrial fibrillation subtypes and its relation with clinical risk factors. Clin Res Cardiol.

[54] Watanabe T, Takeishi Y, Hirono O (2005). C-reactive protein elevation predicts the occurrence of atrial structural remodeling in patients with paroxysmal atrial fibrillation. Heart Vessels.

[55] Psychari SN, Apostolou TS, Sinos L, Hamodraka E, Liakos G, Kremastinos DT (2005). Relation of elevated C-reactive protein and interleukin-6 levels to left atrial size and duration of episodes in patients with atrial fibrillation. Am J Cardiol.

[56] Narducci ML, Pelargonio G, Dello Russo A (2011). Role of tissue C-reactive protein in atrial cardiomyocytes of patients undergoing catheter ablation of atrial fibrillation: pathogenetic implications. Europace.

[57] Yo CH, Lee SH, Chang SS, Lee MC, Lee CC (2014). Value of high-sensitivity C-reactive protein assays in predicting atrial fibrillation recurrence: a systematic review and meta-analysis. BMJ Open.

[58] Dowod TAHM, Amin AA, Al-Sulaiman A, Ali JI, Reda M (2005). C-reactive protein in paroxysmal lone atrial fibrillation. Pak J Med Sci.

[59] Chung MK, Martin DO, Sprecher D (2001). C-reactive protein elevation in patients with atrial arrhythmias: inflammatory mechanisms and persistence of atrial fibrillation. Circulation.

[60] Perryman MB, Strauss AW, Buettner TL, Roberts R (1983). Molecular heterogeneity of creatine kinase isoenzymes. Biochim Biophys Acta.

[61] Jeppesen KK, Riber SS, Riber LP (2019). Does creatine phosphokinase MB predict long-term cardiac death or atrial fibrillation?. Open J Thorac Surg.

[62] Das UN (2016). Heart-type fatty acid-binding protein (H-FABP) and coronary heart disease. Indian Heart J.

[63] Sbarouni E, Georgiadou P, Chaidaroglou A, Degiannis D, Voudris V (2011). Heart-type fatty acid binding protein in elective cardioversion of atrial fibrillation. Clin Biochem.

[64] Rader F, Pujara AC, Pattakos G (2013). Perioperative heart-type fatty acid binding protein levels in atrial fibrillation after cardiac surgery. Heart Rhythm.

[65] Shingu Y, Yokota T, Takada S (2018). Decreased gene expression of fatty acid binding protein 3 in the atrium of patients with new onset of atrial fibrillation in cardiac perioperative phase. J Cardiol.

[66] Roy D, Quiles J, Sinha M, Aldama G, Gaze D, Kaski JC (2004). Effect of direct-current cardioversion on ischemia-modified albumin levels in patients with atrial fibrillation. Am J Cardiol.

[67] Wilson DP, Jacobson TA, Jones PH (2019). Use of lipoprotein(a) in clinical practice: a biomarker whose time has come. A scientific statement from the National Lipid Association. J Clin Lipidol.

[68] Saeedi R, Frohlich J (2016). Lipoprotein(a), an independent cardiovascular risk marker. Clin Diabetes Endocrinol.

[69] Garg PK, Guan W, Karger AB (2020). Lp(a) (lipoprotein [a]) and risk for incident atrial fibrillation: multi-ethnic study of atherosclerosis. Circ Arrhythm Electrophysiol.

[70] Aronis KN, Zhao D, Hoogeveen RC (2017). Associations of lipoprotein(a) levels with incident atrial fibrillation and ischemic stroke: the ARIC (Atherosclerosis Risk in Communities) study. J Am Heart Assoc.

[71] Mohammadi-Shemirani P, Chong M, Narula S (2022). Elevated lipoprotein(a) and risk of atrial fibrillation: an observational and Mendelian randomization study. J Am Coll Cardiol.

[72] Díaz-Peromingo JA, Albán-Salgado A, García-Suárez F, Sánchez-Leira J, Saborido-Froján J, Iglesias-Gallego M (2006). Lipoprotein(a) and lipid profile in patients with atrial fibrillation. Med Sci Monit.

[73] Igarashi Y, Yamaura M, Ito M, Inuzuka H, Ojima K, Aizawa Y (1998). Elevated serum lipoprotein(a) is a risk factor for left atrial thrombus in patients with chronic atrial fibrillation: a transesophageal echocardiographic study. Am Heart J.

[74] Mamazhakypov A, Sartmyrzaeva M, Sarybaev AS, Schermuly R, Sydykov A (2022). Clinical and molecular implications of osteopontin in heart failure. Curr Issues Mol Biol.

[75] Lutz M, von Ingersleben N, Lambers M (2017). Osteopontin predicts clinical outcome in patients after treatment of severe aortic stenosis with transcatheter aortic valve implantation (TAVI). Open Heart.

[76] Rosenberg M, Zugck C, Nelles M (2008). Osteopontin, a new prognostic biomarker in patients with chronic heart failure. Circ Heart Fail.

[77] Lin R, Wu S, Zhu D, Qin M, Liu X (2020). Osteopontin induces atrial fibrosis by activating Akt/GSK-3β/β-catenin pathway and suppressing autophagy. Life Sci.

[78] Du X, Liu T, Shen C (2022). Anti-fibrotic mechanism of SPP1 knockdown in atrial fibrosis associates with inhibited mitochondrial DNA damage and TGF-β/SREBP2/PCSK9 signaling. Cell Death Discov.

[79] Kuenzel S, Klapproth E, Kuenzel K (2020). PLK2 is a novel regulator of osteopontin-driven fibrosis and diastolic dysfunction in permanent atrial fibrillation. Eur Heart J.

[80] Tipteva TA, Chumakova OS, Reznichenko NE (2017). Serum osteopontin level is associated with presence of atrial fibrillation in calcific aortic valve stenosis. J Clin Practice.

[81] Molvin J, Jujic A, Melander O (2020). Exploration of pathophysiological pathways for incident atrial fibrillation using a multiplex proteomic chip. Open Heart.

[82] Güneş HM, Babur Güler G, Güler E (2017). Relationship between serum osteopontin level and atrial fibrillation recurrence in patients undergoing cryoballoon catheter ablation. Turk Kardiyol Dern Ars.

[83] Pascual-Figal DA, Ordoñez-Llanos J, Tornel PL (2009). Soluble ST2 for predicting sudden cardiac death in patients with chronic heart failure and left ventricular systolic dysfunction. J Am Coll Cardiol.

[84] Dudek M, Kałużna-Oleksy M, Migaj J, Straburzyńska-Migaj E (2020). Clinical value of soluble ST2 in cardiology. Adv Clin Exp Med.

[85] Krittayaphong R, Pumprueg S, Sairat P (2022). Soluble ST2 in the prediction of heart failure and death in patients with atrial fibrillation. Clin Cardiol.

[86] Tan R, Yu H, Han X (2021). Circulating soluble suppression of tumorigenicity 2 predicts recurrence after radiofrequency ablation of persistent atrial fibrillation. Front Cardiovasc Med.

[87] Al-Wahaibi K, Khan I, McAdam BF, Leong T (2021). Both ST2 and BNP predicts atrial fibrillation independently of heart failure but BNP is stronger when used in combination. Circulation.

[88] Okar S, Kaypakli O, Şahin DY, Koç M (2018). Fibrosis marker soluble ST2 predicts atrial fibrillation recurrence after cryoballoon catheter ablation of nonvalvular paroxysmal atrial fibrillation. Korean Circ J.

[89] Wang Z, Cheng L, Zhang J (2020). Serum-soluble ST2 is a novel biomarker for evaluating left atrial low-voltage zone in paroxysmal atrial fibrillation. Med Sci Monit.

[90] Wałek P, Gorczyca I, Grabowska U, Spałek M, Wożakowska-Kapłon B (2020). The prognostic value of soluble suppression of tumourigenicity 2 and galectin-3 for sinus rhythm maintenance after cardioversion due to persistent atrial fibrillation in patients with normal left ventricular systolic function. Europace.

[91] Sun WP, Du X, Chen JJ (2022). Biomarkers for predicting the occurrence and progression of atrial fibrillation: soluble suppression of tumorigenicity 2 protein and tissue inhibitor of matrix metalloproteinase-1. Int J Clin Pract.

[92] Chen L, Chen W, Shao Y (2022). Association of soluble suppression of tumorigenicity 2 with new-onset atrial fibrillation in acute myocardial infarction. Cardiology.

[93] Kong L, Hu P, Li C, Jiang T, Hu G (2020). The correlation between SSt2 and atrial fibrillation and its clinical significance. Yangtze Medicine.

[94] Denysiak J, Krummen DE, Anzenberg P, Hsu JC, Daniels LB (2016). Elevated ST2 is associated with more extensive atrial remodeling and longer ablation procedure duration in patients with atrial fibrillation and flutter. Circulation.

[95] Liu H, Wang K, Lin Y (2020). Role of sST2 in predicting recurrence of atrial fibrillation after radiofrequency catheter ablation. Pacing Clin Electrophysiol.

[96] Ma X, Yuan H, Luan HX, Shi YL, Zeng XL, Wang Y (2018). Elevated soluble ST2 concentration may involve in the progression of atrial fibrillation. Clin Chim Acta.

[97] Chen C, Qu X, Gao Z (2018). Soluble ST2 in patients with nonvalvular atrial fibrillation and prediction of heart failure. Int Heart J.

[98] Sanada S, Hakuno D, Higgins LJ, Schreiter ER, McKenzie AN, Lee RT (2007). IL-33 and ST2 comprise a critical biomechanically induced and cardioprotective signaling system. J Clin Invest.

